# Validity evaluation of the Health Information Preferences Questionnaire among college students

**DOI:** 10.3389/fpubh.2024.1249621

**Published:** 2024-03-27

**Authors:** Kaiqi Tang, Junfeng Yuan, Lin Luo

**Affiliations:** ^1^School of Physical Education, Guizhou Normal University, Guiyang, China; ^2^Guizhou Vocational College of Aviation Technology, Guiyang, China

**Keywords:** health information preference, college students, health behavior and outcomes, Health Information Preference Questionnaire, validity and reliability

## Abstract

**Objective:**

This study aims to explore the association between health information preferences and specific health behaviors and outcomes, such as preventive measures and chronic disease management among college students. It assesses how different levels of health information preference influence individuals’ utilization, perception, and self-efficacy within healthcare and health information contexts. Given the rising prevalence of non-communicable chronic diseases among younger populations in China, this research seeks to understand how tailored health information preferences can support effective health education and behavioral interventions. The development of the Health Information Preference Questionnaire (HIPQ) aims to bridge the existing gap in tools for assessing health information preferences among Chinese college students, with a focus on collecting validity evidence to confirm the HIPQ’s applicability in this group.

**Methods:**

The study employed a mixed-methods approach, beginning with an initial item pool derived from a comprehensive review of existing research tools, literature, and expert inputs. An expert review panel conducted item evaluations, leading to item reduction for clarity and relevance. The validation process utilized two independent samples of college students, detailing the sample size (*n* = 446 for preliminary testing, *n* = 1,593 for validation) and characteristics (age, major, urban vs. rural background) to enhance the understanding of the study’s generalizability.

**Results:**

The HIPQ, comprising 25 items across five dimensions—prevention-oriented approaches, relationship with healthcare providers, self-efficacy in obtaining health information, perception of the importance of health information, and health information behavior—demonstrated excellent content validity (ICVI ranged from 0.72 to 0.86). Factor analysis confirmed significant loadings for each item across the anticipated factors, with fit indices (RMSEA = 0.065, CFI = 0.942) supporting good model fit. The HIPQ’s reliability was underscored by Cronbach’s alpha coefficients (>0.8) for each subscale, with significant correlations across all subscales, indicating strong internal consistency and construct validity.

**Conclusion:**

The HIPQ proves to be a reliable and valid instrument for assessing health information preferences among Chinese college students, highlighting its potential for broader application in health education and intervention strategies. Recognizing the study’s focus on a specific demographic, future research should investigate the HIPQ’s adaptability and utility in broader populations and different cultural settings. The study’s limitations, including its concentrated demographic and context, invite further exploration into the HIPQ’s applicability across diverse groups. Additionally, potential future research directions could include longitudinal studies to assess the impact of tailored health information on actual health outcomes and behaviors.

## 1 Introduction

The 20th century witnessed a shift from infectious to lifestyle-related diseases, underscoring the evolving health challenges and the necessity for adaptive health information dissemination strategies. This historical shift highlights the need for updated health communication tools, particularly for engaging populations like Chinese university students, whose health information needs are influenced by both global trends and local cultural contexts. This study aims to develop a tailored Health Information Preference Questionnaire (HIPQ) to meet these needs. Currently, the primary causes of diseases in modern populations are increasingly linked to everyday life and behaviors. Alongside the change in disease types, the focus of health information communication has gradually shifted from “providing biomedical knowledge” to “promoting behavioral change”.

The advent and proliferation of media channels and emerging information technologies have led to a surge in the volume of accessible health content, enabling consumers to access more health information than ever before. This increased availability of health information can benefit consumers in numerous ways, notably by facilitating more informed decisions regarding health and healthcare (1). Consequently, organizations like the World Health Organization and its member states are intensifying efforts to enhance health information systems, aiming to ensure comprehensive access to health information and address healthcare disparities (2, 3). Policymakers are increasingly viewing informed consumer decision-making as a strategic avenue to reduce healthcare costs (4, 5).

Despite the importance of high-quality health information, not all individuals actively seek or engage in healthcare decision-making (6). Certain groups, particularly those affected by the digital divide, are more inclined to rely on health information from medical professionals (7). Rural immigrant populations often exhibit a preference for face-to-face health information access (8). Additionally, there are consumers who are either averse to utilizing health information in healthcare decisions (9) or prefer not to participate in their healthcare decision-making processes at all (10). Factors like literacy barriers (11), the digital divide (12), misinformation (13), trust in healthcare providers (14), and information overload can significantly influence consumers’ motivation and ability to access and utilize health information (15).

Health information preference encompasses an individual’s experience with the content, format, and motivations for seeking health information (16). Research has highlighted that demographic characteristics, environmental factors, and personal information needs can influence consumers’ health information preferences and their likelihood of seeking health information (17, 18). Several studies have explored consumers’ health information seeking behaviors to understand these preferences. For instance, LaMonica et al. explored the health information preferences of older adults, including technological preferences and barriers, well-being, and facilitators (19). Ramsey et al. examined consumer health information preferences concerning information sources, seeking methods, and topics (20). Such preferences can significantly impact an individual’s health literacy, which in turn affects their health behavior and outcomes (21). The positive influence and significance of health information preference on health behavior and outcomes have been well-documented (22, 23).

While existing tools provide a foundation (24, 25), the HIPQ introduces a novel approach tailored to the digital age and cultural context of Chinese university students, anticipating findings that reveal nuanced preferences and behaviors. These instruments primarily assess individuals’ behaviors and attitudes towards seeking health information, encompassing aspects like channels, frequency, purpose, trustworthiness, and utilization of health information. However, with the rapid evolution of Internet technology, there have been substantial changes in health information channels and dissemination methods. New media health information dissemination has emerged as a crucial method of health communication. The COVID-19 pandemic has drastically altered health information seeking behaviors, with Chinese university students facing unique challenges and shifts in their preferences for health-related content, underscoring the urgency of developing the HIPQ (26, 27). Nonetheless, these existing health information preference measurement tools, developed relatively early, may not align with the current societal context characterized by rapid Internet and information technology development. Cultural nuances significantly impact health perceptions, with Eastern cultures exhibiting unique health beliefs and practices. In China, traditional views on health intertwine with modern health information seeking, necessitating tools that reflect these cultural specificities (28). Health and disease concepts exhibit significant cultural diversity (29), and these measurement tools have predominantly been developed within a Western cultural context. Recognizing the gap in culturally sensitive health information tools, this study specifically targets the development of the HIPQ for Chinese students, bridging the divide with a focus on their unique preferences.

Chinese university students represent a pivotal demographic for health communication research due to their high media literacy and susceptibility to behavioral health influences. This study focuses on this group to understand and address their specific health information preferences. Validity evidence has been gathered for this questionnaire to measure its effectiveness in evaluating health information preferences among Chinese university students.

## 2 Materials and methods

**Figure 1:**
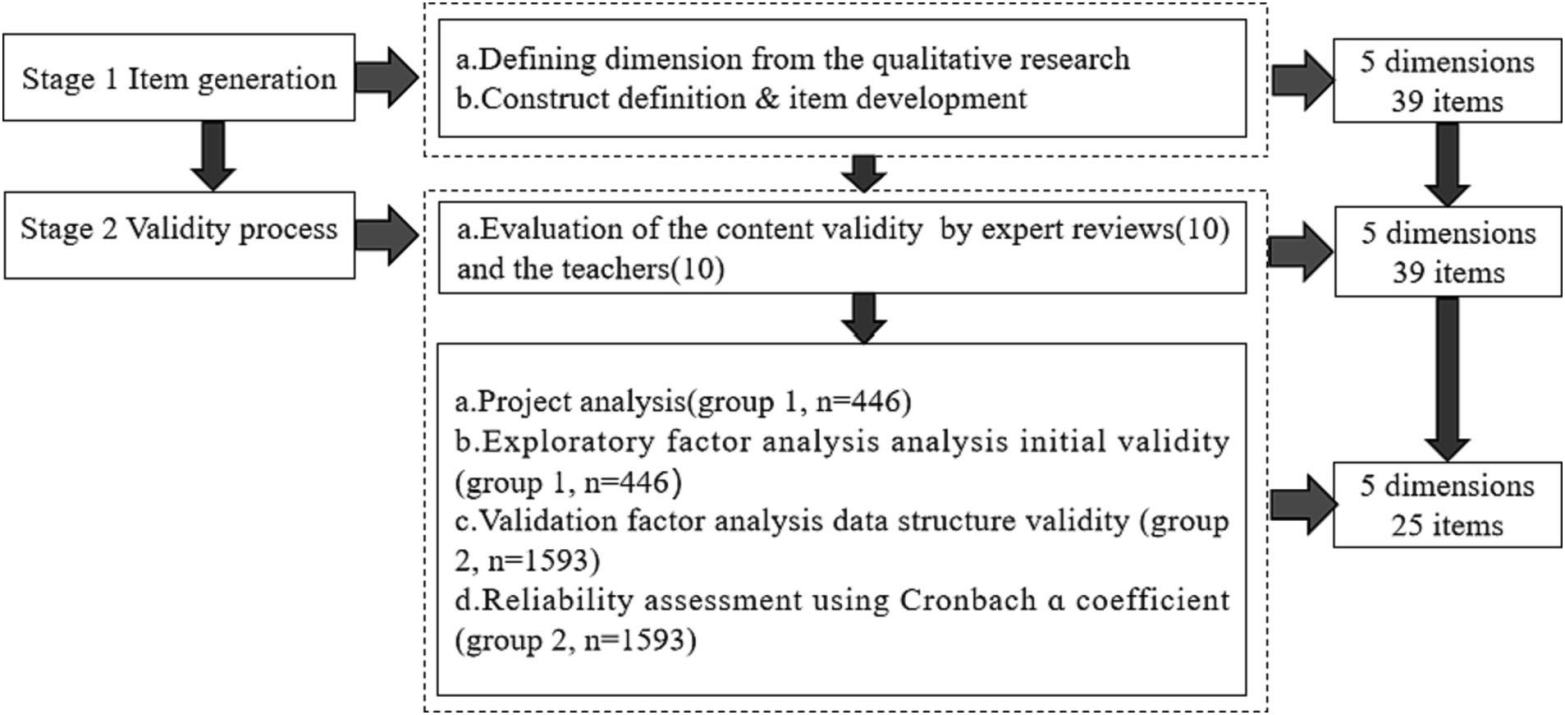
The research process of HIPQ.

The HIPQ was developed in two stages: item generation and validity assessment. This approach streamlined the process into:

- Item generation: We reviewed health information behavior literature, applying social cognitive theory to define preference dimensions.
- Validity assessment: Experts and statistical tests validated the HIPQ’s content and structure, adhering to AERA-APA guidelines (Figure 1) (30, 31).

### 2.1 Item generation

In the process of item generation, we conducted a comprehensive synthesis of the literature on health information behaviors, identifying five critical dimensions of health preference: prevention orientation, relationship with healthcare providers, self-efficacy in obtaining health information, perception of the importance of health information, and health information behavior. Guided by the principles of social cognitive theory (32), this analytical effort culminated in the creation of the preliminary 39-item HIPQ, with each item distinctly mirroring a specific facet of health information preference. The foundational set of indicators, encompassing five dimensions and 39 items, was formulated with contributions from the inaugural expert panel, which comprised three experts each possessing more than a decade of experience in health education and communication. The items were evaluated using a 5-point Likert scale, with scoring options ranging from 1 (strongly disagree) to 5 (strongly agree).

### 2.2 Validity process

For content validity, we engaged a panel of experts, meticulously selected for their authoritative knowledge in health information and behavior. Their diverse expertise, from senior professionals to Ph.D. holders, enriched the HIPQ’s validation process. This process resulted in the selection of nearly 20 experts, with responses received from eight distinguished individuals (six with senior professional titles and two holding Ph.D. degrees). To ensure a unified understanding of the content validity indicators (relevance, clarity, and comprehensiveness), detailed explanations of these concepts were provided to the experts. The content validity assessment questionnaire, comprising both objective and subjective queries, was distributed via email. Objective questions employed a Likert scale format, ranging from 1 (not important at all) to 5 (very important), aiming to evaluate the significance of primary and secondary indicators, as well as their operational observation points. The feedback received was meticulously analyzed and discussed with the first expert group, leading to necessary textual modifications in the item descriptions.

The process was further refined by inviting ten university students to assess the comprehensibility of the questionnaire items. Their feedback highlighted potential comprehension challenges, leading to revisions in the item descriptions for enhanced clarity and accessibility. The revised questionnaire was then sent back to the second expert group for a final evaluation of clarity, relevance, and comprehensiveness, using a rating scale from 1 (completely unreasonable) to 5 (very reasonable). The collected feedback was quantitatively analyzed to estimate the content validity, with an Item Content Validity Index (ICVI) of ≥0.70 (33) indicating acceptable consistency. The overall relevance and clarity of the questionnaire were assessed using the Scale Content Validity Index (SCVI). To calculate the SCVI, the average S-CVI/Ave was computed by summing the ICVIs and dividing by the total number of items.

The comprehensiveness of the questionnaire was ensured by maintaining a consistent number of items throughout the development process. The HIPQ was specifically tailored to the linguistic patterns of Mandarin Chinese speakers, with an estimated completion time of 10–15 min. For internal validity, we applied exploratory factor analysis (EFA) with strict criteria (loading factor > 0.40) and confirmatory factor analysis (CFA) using indices (RMSEA <0.1, CFI > 0.90) to ensure statistical rigor in validating the HIPQ’s structure. Initial data from group 1 were used for item analysis and exploratory factor analysis (EFA), adhering to criteria such as an absolute loading factor > 0.40, average inter-item correlation >0.20, and ensuring no overlap or redundancy in item phrasing (33). This led to a refined HIPQ consisting of 34 items across five domains. Data from group 2 were then employed for confirmatory factor analysis (CFA) to validate the internal structure, using criteria including a root mean square error of approximation (RMSEA) < 0.1 (34), a significant *p*-value with Chi-square/degrees of freedom <5 (35), and a comparative fit index (CFI) > 0.90 (36). Post-model fit, Cronbach’s alpha was used to assess the internal consistency of the entire questionnaire and its five subscales (37). The surveys were conducted between November 2022 and April 2023, utilizing a 5-point Likert scale (1 = completely disagree to 5 = completely agree). All data were processed using SPSS 26.0 and AMOS23.0.

### 2.3 Sampling and sample size

The determination of sample size for each phase of the study adhered to statistical principles and practical considerations. In the development and validation of the HIPQ, the sample sizes for each phase were as follows:

#### 2.3.1 Phase 1: preliminary testing

For the initial survey, the sample size calculation was based on the common recommendation of having 5 to 10 times the number of items in the questionnaire for exploratory factor analysis (EFA). With the HIPQ initially containing 39 items, a minimum sample size of 195 (5 times 39) to 390 (10 times 39) was required. To ensure robustness and accommodate potential non-responses or incomplete questionnaires, the target was set at the upper limit, resulting in 446 valid responses. This size provided a sufficiently diverse sample to test the initial structure of the questionnaire and perform item analysis effectively.

#### 2.3.2 Phase 2: content validity and comprehensiveness assessment

While using snowball sampling for broader reach, we acknowledge its bias potential. We initiated sampling from diverse points to curtail bias, ensuring a more representative demographic spread. Snowball sampling was chosen to reach a broad and diverse population, especially when targeting a demographic that is widely dispersed or not well-defined. Despite its non-random nature, snowball sampling facilitated the recruitment of a sample that might be challenging to reach through traditional methods. To mitigate the inherent biases of this method, the sampling process was initiated from multiple, varied points to enhance sample diversity. A total of 1,593 valid questionnaires were collected, significantly exceeding the initial sample size requirement and offering a comprehensive assessment across different demographics.

#### 2.3.3 Statistical justification and practical considerations

The rationale behind these sample sizes was grounded in statistical theory, complemented by practical considerations like resource availability, time constraints, and accessibility of the target population. These factors were critical in determining the feasibility of collecting a large and diverse sample, especially for the second phase using snowball sampling.

By adhering to these guidelines and acknowledging the limitations and strengths of the chosen sampling method, the study ensured that the sample sizes for both phases were adequate to meet the research objectives. This approach allowed for a thorough evaluation and validation of the HIPQ.

### 2.4 Data collection

The validation of the HIPQ was facilitated by a two-phase data collection approach (Table 1).

**Table 1:** Participant characteristics across two samples.

Description	Phase 1: preliminary survey	Phase 2: expanded survey
Total responses	446	1,593
Gender distribution	244 males (54.71%)	692 males (43.44%)
	202 females (45.29%)	901 females (56.56%)
Average age	Males: 20.32 ± 1.34 years	Males: 19.60 ± 1.29 years
	Females: 19.77 ± 1.37 years	Females: 19.50 ± 1.20 years
Location	Urban: 77 students (17.26%)	Urban: 288 students (18.08%)
	Rural: 369 students (82.74%)	Rural: 1305 students (81.92%)

#### 2.4.1 Phase 1: initial data collection

The first phase was carried out at two universities in the researchers’ city, including a vocational school and an undergraduate university. An electronic questionnaire was distributed through the “Maike” electronic questionnaire platform to facilitate the survey process. This phase successfully gathered 446 valid responses, meeting the methodological requirement of having 5–10 times the number of questionnaire items for a robust analysis. The demographic profile revealed a gender distribution of 244 male students (54.71%), averaging 20.32 ± 1.34 years, and 202 female students (45.29%), averaging 19.77 ± 1.37 years. The majority of participants, 369 individuals (82.74%), were from rural backgrounds, while urban students accounted for 77 responses (17.26%).

#### 2.4.2 Phase 2: expanded data collection

The second phase employed snowball sampling to reach undergraduate students across the eastern, central, and southwestern regions of China. This method enabled a more diverse and extensive data collection, resulting in 1593 valid responses. The gender distribution in this phase indicated higher female participation, with 901 female students (56.56%) averaging 19.50 ± 1.20 years, and 692 male students (43.44%) averaging 19.60 ± 1.29 years. As in the first phase, the majority of participants, 1,305 students (81.92%), were from rural households, with urban households comprising 288 responses (18.08%).

This two-step data collection strategy yielded a comprehensive and diverse dataset, critical for the rigorous validation of the HIPQ across various demographic segments.

## 3 Results

### 3.1 Item generation

An extensive review of the literature on health information preferences, coupled with inputs from a panel of three field experts, led to the identification of five primary dimensions of health information preferences. These dimensions are: (1) Prevention-Oriented Approaches, focusing on preventive measures and proactive health management; (2) Relationship with Healthcare Providers, emphasizing patient-provider communication and trust; (3) Self-Efficacy in Obtaining Health Information, reflecting an individual’s confidence in accessing and understanding health-related data; (4) Perceived Importance of Health Information, indicating the value individuals place on such information; and (5) Health Information Behavior, encompassing actions based on received health information.

To enhance the study, these primary dimensions were expanded upon by developing a comprehensive set of secondary indicators. These indicators were operationalized through a rigorous methodology, which involved a systematic approach to measuring and evaluating each aspect of the primary dimensions. The process included establishing selection criteria for indicators, developing measurement scales, and testing for reliability and validity.

The result is the evaluation indicator system detailed in this study, offering a novel and comprehensive framework for understanding health information preferences. Presented in Table 2, this system provides a robust structure for assessing various aspects of health information preferences.

**Table 2:** HIPQ evaluation index system.

Initial dimension	Secondary indicators	Coding	Item
Prevention-oriented	The importance of healthy living	Q1	Living the healthiest life possible is very important to me
	The importance of measures such as diet and exercise	Q2	Proper diet, exercise, and preventive measures help me stay healthy
	The importance of longevity	Q3	Longevity is important to me
	The importance of physical condition to health	Q4	My health status depends on my physical condition
	Self-care ability	Q5	I will take good care of myself
	Attention to health risks	Q6	I try to understand my personal health risks
	Efforts in disease prevention	Q7	I actively work to prevent diseases
	Efforts in maintaining health	Q8	I do my best to maintain my health
	Control in maintaining health	Q9	I wish I could have more control over my own health
Relationship with healthcare provider	Relying on doctors for health decisions	Q10	I let the doctor make the right decisions about my health
	Acquiring health management knowledge from doctors	Q11	I rely on the doctor to tell me everything I need to know about managing my health
	Doctors as sources of health information	Q12	My doctor is a good source of information on health issues
	Doctors assisting in health management	Q13	I work with my doctor to manage my health
	Analyzing health information with doctors	Q14	When I read or hear something related to my health, I tell my doctor about it
	Communicating with doctors promptly	Q15	I make sure the doctor answers my questions in a way I can understand
	Relationship with healthcare providers	Q16	I have a good relationship with my healthcare provider
	Understanding doctors’ language	Q17	I often do not understand the language my doctor uses
	Practicality of health information provided by doctors	Q18	My doctor provides me with practical health information
	Discussing treatment plans with doctors	Q19	Before deciding on a treatment plan, I will discuss all possible treatment options with my doctor
Self-efficacy for obtaining health information	The effectiveness of seeking health information	Q20	I can find good health information
	Confidence in obtaining answers to health issues	Q21	I have never found a good answer to my health problems
	Preferences for health issues	Q22	I like to learn about health issues
	Fears of health issues	Q23	Most health problems are too complicated for me to understand
	Fear of health information	Q24	I am overwhelmed by the massive amount of health information
	Difficulty in understanding health information	Q25	It is difficult for me to understand the health information I read
Perception of health information’s importance	Scenario-based needs for health issues	Q26	I would like to have more video programs that incorporate storylines related to health issues
	Understanding the importance of health issues	Q27	Understanding health issues is very important
	Evaluation of media health reports	Q28	The media has spent too much time reporting on health issues
	The importance of health information	Q29	To maintain good health, it is crucial to understand health information
	The importance of sources of health information	Q30	I feel much better if I can verify health advice from multiple sources
	The importance of health information for individual health	Q31	The abundance of available health information makes it easier for me to take care of my health
	The importance of health information for family health	Q32	I need to know about health issues so that I can keep myself and my family healthy
Health information behavior	Time for learning about health information	Q33	I do not have time to learn a lot of health information
	Time for reading health information	Q34	I want to read or watch stories about health
	Attention to health information	Q35	I do not pay attention to health information unless it’s about a problem I have
	Access to disease information	Q36	When I’m sick, I try to get information about my illness
	Diversity of channels for obtaining health information	Q37	I like to get health information from various sources.
	Access to medication information	Q38	When I take medication, I try to get as much information as possible about the benefits and side effects
	Making decisions about health	Q39	Before deciding on my health status, I will do my best to address the issue

Items with bold numbers are scored in reverse.

### 3.2 Validity process

#### 3.2.1 Expert review and response process

The Item Content Validity Index (ICVI) for the initial set of items varied between 0.72 and 0.86, indicating a substantial level of expert agreement on the appropriateness and relevance of the selected items and their corresponding questions. Experts evaluated the items for relevance to the study objectives, clarity in wording, and comprehensiveness in covering the dimensions of health information preferences. The concordance rates for these aspects were 80.22% for relevance, 76.77% for clarity, and 82.11% for comprehensiveness, demonstrating high consistency across expert evaluations.

Additionally, ten college student volunteers participated in a pilot testing phase to assess the questionnaire’s accessibility and understandability. Their feedback led to modifications in the clarity of the language, particularly for questions 26 and 29, to ensure comprehensibility for a wider audience.

Regarding the scoring mechanism, eight items (Q17, Q21, Q23, Q24, Q25, Q28, Q33, and Q35) were designated for reverse scoring to mitigate potential response biases and enhance data analysis robustness. The rest of the items were scored in a positive direction. The rationale for selecting specific items for reverse scoring was to mitigate response bias and enhance the tool’s sensitivity to nuanced health information preferences. This decision was based on item content that conceptually opposes the dimension’s core theme, ensuring a balanced approach to measuring each dimension.

Each item’s content, along with their respective scoring directions, is detailed in Table 2. This thorough presentation provides clear insights into the HIPQ’s structure and focus areas, enhancing its applicability in diverse research settings.

#### 3.2.2 Internal structural analysis

##### 3.2.2.1 Project analysis

In this study, SPSS 26.0 software was used to conduct a detailed analysis of the basic characteristics of the measurement items from the initial survey data of the HIPQ. Table 3 presents a statistical summary of these characteristics for the 39 items, including mean, standard deviation, skewness, and kurtosis (Table 3).

**Table 3:** Results of item analysis for HIPQ (*n* = 446).

Coding	Mean	SD	Skewness	Peak	CR	Correlation coefficient with total scale
Q1	3.946	0.862	−0.636	0.491	9.780^**^	0.545^**^
Q2	4.020	0.844	−0.895	1.399	8.016^**^	0.524^**^
Q3	3.814	0.904	−0.633	0.477	11.128^**^	0.554^**^
Q4	3.861	0.820	−0.622	0.707	10.563^**^	0.580^**^
Q5	3.989	0.775	−0.680	1.211	8.805^**^	0.583^**^
Q6	3.863	0.836	−0.830	1.404	10.415^**^	0.626^**^
Q7	3.993	0.772	−0.607	0.896	10.355^**^	0.643^**^
Q8	4.034	0.738	−0.626	1.063	9.598^**^	0.641^**^
Q9	4.013	0.776	−0.777	1.431	9.377^**^	0.634^**^
Q10	3.836	0.844	−0.876	1.363	10.920^**^	0.598^**^
Q11	3.646	0.917	−0.541	0.228	10.405^**^	0.497^**^
Q12	3.800	0.809	−0.619	0.887	11.458^**^	0.608^**^
Q13	3.664	0.874	−0.835	1.073	13.478^**^	0.618^**^
Q14	3.632	0.884	−0.720	0.643	14.768^**^	0.625^**^
Q15	3.769	0.803	−0.603	0.752	16.216^**^	0.684^**^
Q16	3.670	0.885	−0.633	0.381	11.271^**^	0.526^**^
Q17	3.415	0.965	−1.378	1.426	1.947	0.049
Q18	3.756	0.805	−0.801	1.369	13.553^**^	0.638^**^
Q19	3.713	0.809	−0.762	1.318	13.879^**^	0.612^**^
Q20	3.809	0.723	−0.625	1.355	15.170^**^	0.715^**^
Q21	3.487	0.966	−1.463	1.715	2.410^*^	0.081
Q22	3.796	0.744	−0.769	1.912	12.027^**^	0.646^**^
Q23	3.424	0.952	−1.455	1.591	4.369^**^	0.187^**^
Q24	3.424	0.930	−1.578	1.817	2.765^**^	0.103^*^
Q25	3.491	0.886	−1.539	2.319	2.810^**^	0.126^*^
Q26	3.648	0.820	−0.736	0.979	13.472^**^	0.527^**^
Q27	3.899	0.759	−0.572	0.988	10.408^**^	0.634^**^
Q28	3.428	0.942	−1.548	1.733	1.770	0.034
Q29	3.921	0.734	−0.878	2.342	10.792^**^	0.625^**^
Q30	3.812	0.782	−0.902	1.884	10.883^**^	0.642^**^
Q31	3.852	0.762	−0.786	1.679	10.891^**^	0.645^**^
Q32	3.854	0.755	−0.760	1.710	12.235^**^	0.666^**^
Q33	3.433	0.955	−1.520	1.652	2.986^**^	0.090
Q34	3.489	0.980	−1.479	1.655	2.339^*^	0.090
Q35	3.805	0.747	−0.513	0.953	10.040^**^	0.577^**^
Q36	3.899	0.741	−0.702	1.621	9.720^**^	0.583^**^
Q37	3.825	0.741	−0.574	0.941	10.144^**^	0.564^**^
Q38	3.794	0.789	−0.803	1.613	10.779^**^	0.545^**^
Q39	3.787	0.780	−0.921	2.021	11.150^**^	0.544^**^

^**^p < 0.01.

The skewness and kurtosis values for each of the 39 items were carefully evaluated, ensuring that their absolute values were <2. This threshold, as suggested by Polit and Beck (33), indicates a normal distribution of responses, confirming that the collected data did not exhibit significant deviations from normality.

The results, specifically focusing on the critical ratio (CR) values, are detailed in Table 3. The analysis showed that, with the exception of items Q17, Q21, Q28, Q33, and Q34, the CR values for all other items achieved statistical significance at *p* < 0.01. Moreover, these items demonstrated a meaningful correlation with the total score, with correlation coefficients exceeding 0.20, indicating strong discriminative ability. Based on the analysis results, items Q17, Q21, Q28, Q33, and Q34 were removed, reducing the total number of items to 34.

Adhering to the methodological recommendations of Polit and Beck (33), two-sample *t*-tests were conducted to examine the differences in responses for each item between the two groups. This statistical approach is crucial for assessing the efficacy of each item in distinguishing responses effectively.

The results of these tests were significant: all 34 items displayed sufficient discriminative power (Table 4). This critical improvement resulted in the development of a more streamlined and effective questionnaire, now consisting of 34 items. Each retained item exhibited strong discriminative ability, significantly bolstering the overall robustness and reliability of the tool.

**Table 4:** Item analysis (discrimination) analysis results (*n* = 446).

Coding	Group (Mean ± SD)		*t*	*p*
	Low score group (*n* = 120)	High score group (*n* = 120)		
Q1	3.03 ± 0.94	4.47 ± 0.81	27.596	<0.001
Q2	3.10 ± 0.92	4.53 ± 0.70	29.52	<0.001
Q3	2.96 ± 0.90	4.38 ± 0.84	27.337	<0.001
Q4	3.00 ± 0.90	4.36 ± 0.79	27.135	<0.001
Q5	3.04 ± 0.86	4.49 ± 0.65	32.046	<0.001
Q6	2.93 ± 0.81	4.42 ± 0.67	33.688	<0.001
Q7	3.05 ± 0.84	4.56 ± 0.57	35.412	<0.001
Q8	3.04 ± 0.87	4.55 ± 0.59	34.35	<0.001
Q9	3.10 ± 0.86	4.57 ± 0.57	34.051	<0.001
Q10	2.99 ± 0.85	4.41 ± 0.71	30.603	<0.001
Q11	2.89 ± 0.78	4.02 ± 1.04	20.635	<0.001
Q12	2.91 ± 0.79	4.28 ± 0.79	29.186	<0.001
Q13	2.83 ± 0.79	4.16 ± 0.91	26.032	<0.001
Q14	2.78 ± 0.80	4.06 ± 0.97	24.183	<0.001
Q15	2.91 ± 0.82	4.26 ± 0.78	28.253	<0.001
Q16	2.93 ± 0.86	4.15 ± 0.89	23.424	<0.001
Q18	2.97 ± 0.87	4.26 ± 0.74	26.775	<0.001
Q19	2.88 ± 0.83	4.26 ± 0.78	28.812	<0.001
Q20	2.92 ± 0.80	4.31 ± 0.68	31.508	<0.001
Q22	2.95 ± 0.85	4.27 ± 0.69	28.767	<0.001
Q23	3.03 ± 0.82	2.81 ± 1.29	3.437	0.001
Q24	2.99 ± 0.86	2.71 ± 1.26	4.238	<0.001
Q25	3.02 ± 0.81	2.88 ± 1.27	2.152	0.032
Q26	2.91 ± 0.79	3.90 ± 0.99	18.54	<0.001
Q27	3.01 ± 0.86	4.43 ± 0.69	30.409	<0.001
Q29	3.02 ± 0.82	4.38 ± 0.69	30.346	<0.001
Q30	2.97 ± 0.81	4.30 ± 0.68	29.863	<0.001
Q31	2.96 ± 0.83	4.33 ± 0.67	30.188	<0.001
Q32	3.03 ± 0.81	4.36 ± 0.68	29.832	<0.001
Q35	3.05 ± 0.84	4.19 ± 0.75	24.158	<0.001
Q36	3.04 ± 0.82	4.33 ± 0.66	29.145	<0.001
Q37	3.02 ± 0.82	4.25 ± 0.75	26.186	<0.001
Q38	3.00 ± 0.86	4.26 ± 0.74	26.518	<0.001
Q39	3.00 ± 0.85	4.28 ± 0.69	27.711	<0.001

##### 3.2.2.2 Exploratory factor analysis

Using the KMO and Bartlett’s Test for validity verification, as seen in the above table: the KMO value is 0.941, which is greater than 0.6, satisfying the prerequisite for factor analysis, indicating that the data are suitable for factor analysis research. Additionally, the data passed Bartlett’s Test of Sphericity (*p* < 0.05), suggesting that the study data are appropriate for factor analysis (Table 5).

**Table 5:** KMO and Bartlett’s test.

KMO value		0.941
Bartlett’s test of sphericity	Approximate Chi-square	8720.47
	*df*	465
	*p*-value	<0.001

To thoroughly assess the internal structure of the Health Information Processing Questionnaire (HIPQ), the study employed the Varimax rotation technique, chosen for its effectiveness in clarifying factor loadings. The exploratory factor analysis (EFA), outlined in Table 6, analyzed 34 individual items. Utilizing a combination of variance contribution rate and Scree Plot analysis, five factors were identified, each with eigenvalues exceeding the threshold of 1. After rotation, these factors accounted for variance explanation rates of 16.058, 14.943, 12.151, 10.575, and 10.198%, cumulatively representing 63.925% of the variance. Importantly, all factor loadings showed absolute values greater than 0.40, indicating strong construct validity. However, a detailed review indicated that items Q1, Q2, Q10, Q11, Q18, Q19, Q20, Q22, and Q26 had comparable cross-factor loadings, suggesting potential issues of construct redundancy or conceptual overlap.

**Table 6:** Explained variance.

Factor number	Before rotation		After rotation	
	Eigenvalues	Variance explained %	Eigenvalues	Variance explained %
1	12.923	41.688	4.978	16.058
2	2.533	8.17	4.632	14.943
3	2.128	6.863	3.767	12.151
4	1.228	3.96	3.278	10.575
5	1.006	3.245	3.161	10.198

In light of these findings, a decision was made to remove these nine items from the questionnaire. A subsequent EFA on the revised item set again identified five factors, each with eigenvalues over 1. This reanalysis resulted in variance explanation rates of 16.921, 16.614, 16.142, 10.893, and 3.961%, slightly increasing the cumulative variance explanation rate to 64.531%. This improvement in the model’s explanatory capability also enhanced the questionnaire’s precision and practical utility. The factor loading coefficients after rotation confirmed the HIPQ’s solid structural validity (Table 7). The factors were labeled as “Prevention-Oriented,” “Relationship with Healthcare Provider,” “Self-Efficacy for Obtaining Health Information,” “Perception of Health Information’s Importance,” and “Health Information Behavior.” As a result, the final structure of the HIPQ encompasses an assessment of 25 items across these five carefully defined dimensions.

**Table 7:** HIPQ exploratory factor analysis results (*n* = 1,593).

*χ*^2^/*df*	GFI	AGFI	IFI	TLI	CFI	RMR	SRMR	RMSEA (90% CI)
7.695	0.942	0.901	0.942	0.934	0.942	0.044	0.048	0.065 (0.058 ~ 0.068)

##### 3.2.2.3 Confirmatory factor analysis

**Figure 2:**
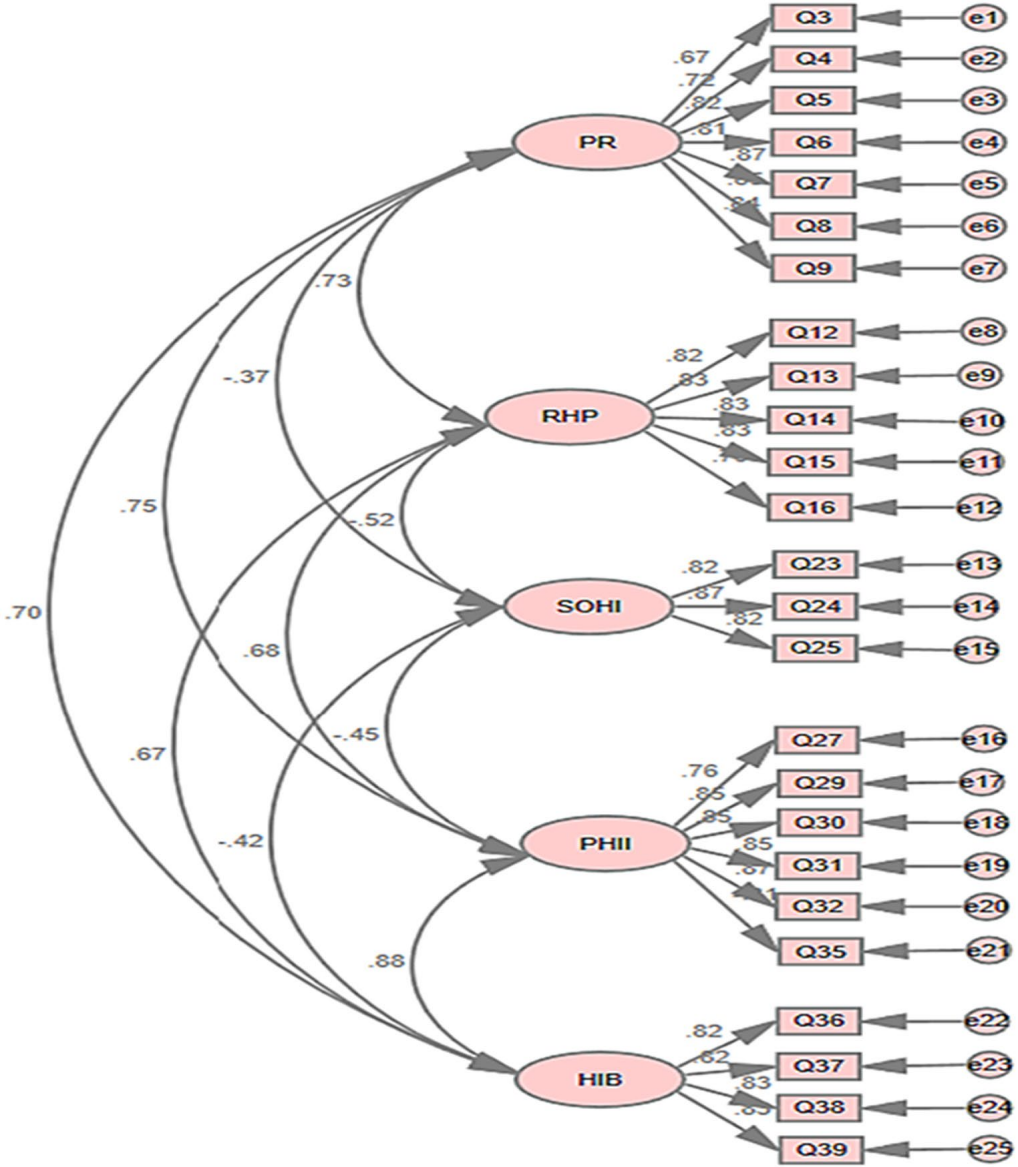
The measurement model of HIPQ. PO, prevention-oriented; RHP, relationship with healthcare provider; SOHI, self-efficacy for obtaining health information; PHII, perception of health information’s importance; HIB, health information behavior.

To verify the stability of the content structure of the Health Information Processing Questionnaire (HIPQ), this study conducted a Confirmatory Factor Analysis (CFA) test using AMOS 23.0 on the data from the second group (*n* = 1,593). The evaluation model incorporated 25 HIPQ items as significant variables, forming five first-order factor latent variables: Prevention-Oriented (7 items), Relationship with Healthcare Providers (5 items), Self-Efficacy for Obtaining Health Information (3 items), Perception of Health Information’s Importance (6 items), and Health Information Behavior (4 items). The maximum likelihood method was employed for the factor validation analysis. The fit indices of the model are displayed in Table 8. The data analysis results indicated that the model possesses good structural validity (see Figure 2). The factor loadings for all items were >0.6, demonstrating that each factor exhibits strong convergent validity.

**Table 8:** Reliability results of the HIPQ.

Factors	Items	Numbers	Cronbach’s α
Prevention-oriented	Q3, Q4, Q5, Q6, Q7, Q8, Q9	7	0.923
Relationship with healthcare provider	Q12, Q13, Q14, Q15, Q16	5	0.911
Self-efficacy for obtaining health information	Q23, Q24, Q25	3	0.891
Perception of health information’s importance	Q27, Q29, Q30, Q31, Q32, Q35	6	0.894
Health information behavior	Q36, Q37, Q38, Q39	4	0.888
HIPQ total		25	0.904

#### 3.2.3 Reliability analysis

SPSS 26.0 software was used to conduct statistical analysis on the Cronbach’s alpha coefficients of the five sub-scales and the total questionnaire. The Cronbach’s alpha coefficients are shown in Table 9, and the Cronbach’s alpha coefficients for all sub-scales and the total questionnaire were approximately 0.8, indicating good reliability of the questionnaire.

**Table 9:** HIPQ exploratory factor analysis results (*n* = 446).

Coding	Factor loading factor					Commonality
	Factor 1	Factor 2	Factor 3	Factor 4	Factor5	
Q3	0.547					0.423
Q4	0.694					0.564
Q5	0.751					0.625
Q6	0.747					0.665
Q7	0.778					0.711
Q8	0.751					0.689
Q9	0.795					0.726
Q12			0.742			0.7
Q13			0.803			0.723
Q14			0.815			0.765
Q15			0.775			0.754
Q16			0.673			0.568
Q23					0.892	0.813
Q24					0.885	0.808
Q25					0.890	0.827
Q27		0.665				0.608
Q29		0.785				0.733
Q30		0.676				0.664
Q31		0.760				0.733
Q32		0.806				0.805
Q35		0.569				0.504
Q36				0.648		0.659
Q37				0.737		0.697
Q38				0.783		0.75
Q39				0.789		0.721

## 4 Discussion

This study delves into the structure and validity of the Health Information Processing Questionnaire (HIPQ), aiming to comprehensively capture individuals’ preferences and behaviors in processing health information. Through an exhaustive literature review, supplemented by insights from three domain experts, we identified five key dimensions of health information preferences: Prevention-Oriented Approaches, Relationship with Healthcare Providers, Self-Efficacy in Obtaining Health Information, Perceived Importance of Health Information, and Health Information Behavior. These dimensions underscore the multifaceted nature of health information processing and lay a robust theoretical foundation for further exploration and practical application.

When comparing our research findings with similar studies, it is noteworthy that the dimensions identified in the HIPQ closely align with those discovered in existing research on health information preferences. For instance, the “Prevention-Oriented Approaches” dimension corresponds to previous studies emphasizing the significance of preventive health behaviors and information-seeking. A study conducted by Johnson et al. in a Western context also emphasized the dimension of prevention-focused health information preferences, indicating cross-cultural relevance (38).

Moreover, the “Relationship with Healthcare Providers” dimension underscores the significance of communication and trust, offering a pathway for HIPQ’s application in healthcare settings. It can be utilized to assess and enhance patient education and health information dissemination strategies, for instance, by tailoring information to meet patient preferences or emphasizing the importance of preventive orientations and self-efficacy in patient-provider communications. Studies conducted in diverse cultural backgrounds, including those by Smith et al. and Chen et al. have consistently highlighted the central role of healthcare provider relationships in shaping health information preferences (39, 40). This cross-cultural consistency suggests that the “Relationship with Healthcare Providers” dimension may be a universal aspect of health information processing.

Although this study did not perform a retest reliability analysis, this limitation could impact the findings’ stability and reliability over time. Future research should consider implementing longitudinal measures to assess the HIPQ’s stability, providing deeper insights and enhancing its credibility for long-term application.

Internal structural analysis, using EFA and CFA, identified five distinct HIPQ dimensions. Notably, some items exhibited cross-factor loadings, suggesting potential construct overlap. We addressed this by refining item definitions and removing or revising items with significant overlap to ensure each dimension’s distinctiveness. The factor loadings, representing how well each item correlates with its underlying dimension, demonstrated strong convergent validity (CR values >0.7). Convergent validity, confirmed by high CR values and significant eigenvalues (indicating the variance captured by a factor), ensures that the dimensions accurately reflect the constructs they are intended to measure (41, 42). Our factor analysis led to the removal of certain items, a decision aimed at enhancing the HIPQ’s construct clarity. This refinement process, while necessary for the tool’s precision, invites further investigation into the removed items’ potential impact on the overall construct and utility.

While this study validates the HIPQ in a Chinese university context, its framework allows for adaptation to diverse demographic groups and cultural settings. Future research should explore its validation and adaptation across various cultures and populations, enhancing its relevance and applicability globally. We incorporated culturally relevant items and adjusted phrasing to resonate with Chinese students’ perspectives on health, demonstrating the tool’s potential adaptability for diverse cultural settings.

The identified interconnections among the HIPQ dimensions hint at potential higher-order factors, such as “comprehensive health information processing capability.” Future studies are encouraged to delve into these higher-order constructs, offering a more integrated understanding of health information preference structures and their implications.

Given the variations in health and disease perceptions across cultures, future studies should provide specific recommendations or considerations for the HIPQ’s adaptations. This could involve incorporating culturally sensitive items or adjusting the phrasing to resonate with different cultural perspectives on health. Future investigations should assess the validity and reliability of the HIPQ in diverse cultural settings to determine its suitability as a global tool for assessing health information preferences.

While focusing on Chinese college students, our sample’s diversity, including age, major, and urban vs. rural background, offers insights into the HIPQ’s generalizability. Future research should aim to include a broader demographic to enhance these findings’ applicability.

The HIPQ’s potential application in health informatics, medicine, and health education is vast. Future work could offer examples or scenarios where HIPQ proves especially beneficial, such as in designing patient-centric health apps, enhancing medical curriculum, or informing public health campaigns. These limitations underscore the need for cautious generalization of the results.

## 5 Conclusion

While the HIPQ demonstrates valid content, response processes, and internal structure, explicit comparisons with existing health information preference tools could further highlight its unique contributions. Future studies should delineate how HIPQ complements or advances the current understanding of health information preferences. Moreover, its practical application in various healthcare settings can significantly enhance the understanding and improvement of health information dissemination strategies, ultimately benefiting patient education and engagement.

## Data availability statement

The raw data supporting the conclusions of this article will be made available by the authors, without undue reservation.

## Ethics statement

This study has been approved by the Ethics Committee of Guizhou Normal University. The studies were conducted in accordance with the local legislation and institutional requirements. Written informed consent for participation was not required from the participants or the participants’ legal guardians/next of kin in accordance with the national legislation and institutional requirements (protocol number GZNUPEI 20220524).

## Author contributions

KT participated in data analysis and manuscript writing. LL contributed to the study design, manuscript drafting, data reduction, analysis, and manuscript editing. JY participated in data collection. All authors contributed to the article and approved the submitted version.
